# Effect of acid-alkali treatment on serum protein adsorption and bacterial adhesion to porous titanium

**DOI:** 10.1007/s10856-022-06646-7

**Published:** 2022-02-02

**Authors:** Juan Zhong, Xuelian Li, Yitong Yao, Jing Zhou, Shanshan Cao, Xinping Zhang, Yutao Jian, Ke Zhao

**Affiliations:** 1 Hospital of Stomatology, Guanghua School of Stomatology, Sun Yat-sen University, Guangdong Provincial Key Laboratory of Stomatology, Guangzhou, China; 2 Guangzhou Yuexiu Stomatological Hospital, Guangzhou, China; 3 School of Materials Science and Engineering, South China University of Technology, Guangzhou, China; 4 Institute of Stomatological Research, Guangdong Provincial Key Laboratory of Stomatology, Sun Yat-sen University, Guangzhou, China

## Abstract

Modification of the titanium (Ti) surface is widely known to influence biological reactions such as protein adsorption and bacterial adhesion in vivo, ultimately controlling osseointegration. In this study, we sought to investigate the correlation of protein adsorption and bacterial adhesion with the nanoporous structure of acid-alkali-treated Ti implants, shedding light on the modification of Ti implants to promote osseointegration. We fabricated nontreated porous Ti (NTPT) by powder metallurgy and immersed it in mixed acids and NaOH to obtain acid-alkali-treated porous Ti (AAPT). Nontreated dense sample (NTDT) served as control. Our results showed that nanopores were formed after acid-alkali treatment. AAPT showed a higher specific surface area and became much more hydrophilic than NTPT and NTDT (*p* < 0.001). Compared to dense samples, porous samples exhibited a lower zeta potential and higher adsorbed protein level at each time point within 120 min (*p* < 0.001). AAPT formed a thicker protein layer by serum precoating than NTPT and NTDT (*p* < 0.001). The main adsorbed proteins on AAPT and NTPT were albumin, α1 antitrypsin, transferrin, apolipoprotein A1, complement C3 and haptoglobin α1 chain. The amounts of bacteria adhering to the serum-precoated samples were lower than those adhering to the nonprecoated samples (*p* < 0.05). Lower-molecular-weight proteins showed higher affinity to porous Ti. In conclusion, acid-alkali treatment facilitated protein adsorption by porous Ti, and the protein coating tended to prevent bacteria from adhering. These findings may be utilized for Ti implant modification aimed at reducing bacterial adhesion and enhancing osseointegration.

**Figure:**
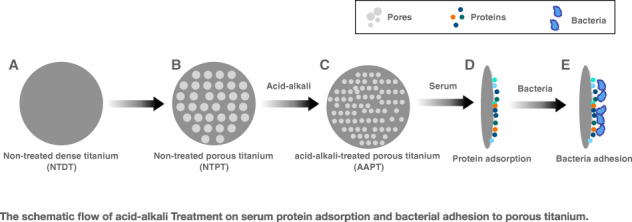
Graphical abstract

Graphical abstract

## Introduction

Ti and its alloys have been widely used in the dental field due to their superior mechanical properties, corrosion resistance and biocompatibility [1]. The success of implantation is determined by osseointegration. Rapid adsorption of protein onto implant surfaces upon contact with blood is the trigger event after implant placement [2, 3]. The adsorbed protein mediates and influences subsequent biological reactions that ultimately control osseointegration [4]. Thus, a thorough understanding of the protein adsorption mechanism between blood and Ti implant surfaces is necessary to predict biological behavior and to guide modification of the implant.

Protein adsorption in vivo is complex and dynamic and is driven by noncovalent interactions, including hydrogen bonding, electrostatic forces, hydrophobic interactions and van der Waals forces [5]. The type, amount and conformation/orientation of adsorbed proteins are largely controlled by the implant surface properties, including surface topography, roughness, chemistry, wettability and charge [2]. Studies on the interactions between one or several specific proteins and biomaterials are common. However, blood contains thousands of proteins that vary in size, concentration and function. Many proteins are involved in mediating cell adhesion, proliferation and differentiation. A variety of proteins from blood or serum are competitively adsorbed on surfaces to form a composite layer when a biomaterial is implanted [6].

The adsorption sequence of competitive proteins has been studied, and it has been suggested that low-molecular-weight proteins arriving first at the surface are generally replaced by high-MW proteins. Albumin replaces adsorbed fibrinogen and fibronectin on TiO_2_, and the released fibrinogen and fibronectin are then adsorbed onto the albumin [7]. On nontreated Ti, acid-etched Ti and anodized Ti, the adsorbed proteins are mainly albumin, transferrin, apolipoprotein A, α1 antitrypsin and vitamin D-binding protein [8]. Accordingly, the competitive adsorption and effects of the proteins should be included in related studies on surface modification of Ti implants.

*Streptococcus* and *Actinomyces* are the two genera that colonize early during biofilm formation and development [9]. The adsorbed protein on implants may mediate bacterial adhesion, resulting in peri-implantitis and implant failure [10]. Implant-associated infection has a significant impact on short- and long-term survival [11, 12]. The initial proteic interface interacts not only with the various host proteins but also with bacteria for adhesion [13]. It has been suggested that bacteria adhere preferentially to rougher and more porous Ti surfaces [14], the nanostructure of which facilitates bacterial biofilm formation [15]. However, the adsorbed protein accumulates and fills in the textured surface, making it less rough, consequently reducing bacterial adhesion [16]. The effect of the adsorbed protein is deemed to be specific. Albumin reduces the adhesion of *Streptococcus mutans* but not *Porphyromonas gingivalis* or *Fusobacterium nucleatum*, while in comparison, fibronectin may enhance the adhesion of both *P. gingivalis* and *F. nucleatum* [17].

The nanostructure has been suggested to be the crucial factor facilitating osseointegration [18–20]. The question arises regarding the extent to which the nanoporous structure of the Ti implant can favor host protein adsorption and cell adhesion, as well as whether successive proteic interactions can competitively and simultaneously suppress bacterial adhesion.

In this study, we aimed to (1) develop and characterize the surface of Ti discs with different modifications and (2) investigate protein adsorption and bacterial adhesion on the surface of acid-alkali-treated Ti implants, shedding light on the modification of Ti implants to promote osseointegration.

## Materials and methods

### Sample preparation

Porous Ti samples were fabricated by powder metallurgy. Briefly, commercially available pure Ti powder (50 µm, Baoji Titanium Industry, China) and highly pure NH_4_HCO_3_ powder (from 0 to 200 µm, 30 wt%, Damao Industry, China), which served as the temporary space holder, were sufficiently blended in a V-blender for 20 h and subsequently cold compacted into cylindrical samples (16 mm × 20 mm, ∅ × h) using a hydraulic press at a pressure of 100 MPa. The conventional sintering method was used to fabricate porous Ti samples. Dense Ti samples had no NH_4_HCO_3_ powder. Raw sintered Ti samples were machined to obtain a disc form (10 mm in diameter and 1.5 mm in thickness). The samples were ground by means of an automatic polishing machine (Struers, Copenhagen, Denmark) with SiC sandpaper graded from 220, 500 and 1000 to 1500 grit. Acid-alkali-treated porous Ti (AAPT) was prepared as previously described [21]. Briefly, porous samples were first immersed in a solution of 48% H_2_SO_4_ and 18% HCl at 70 °C for 15 min and subsequently in 6 M NaOH solution at 70 °C for 12 h [22]. The samples were divided into three groups, i.e., AAPT, nontreated porous Ti (NTPT) and nontreated dense Ti (NTDT). The samples were cleaned ultrasonically in ethanol and distilled water for 30 min each, and both sides were sterilized under ultraviolet irradiation for 30 min each prior to the biological experiments.

### Surface topography

The surface topography of AAPT, NTPT and NTDT was observed by scanning electron microscopy (SEM, Quanta 200, FEI, The Netherlands). The measurements were performed on three samples for each type of Ti.

### Contact angle

Sample wettability (hydrophilicity and hydrophobicity) was determined by measuring the water contact angle with the sessile-drop test with ultrapure water (OCA20, Dataphysics, Germany). A 2-μl droplet was carefully placed on each sample surface. Images of each droplet were taken every 0.07 s for 10 s (a total of ~140 images per sample). The magnified side view of the droplet captured at 4 s was immediately photographed for measurement of the contact angle. Parameters of the samples (*n* = 6) were noted.

### Specific surface area

Nitrogen adsorption was performed by means of a specific surface area (SSA) and pore size analyzer (Autosorb iQ2 MP, Quantachrome, USA). SSAs of samples (*n* = 3) were determined by the Brunauer–Emmett–Teller method.

### Zeta potential

Zeta potential (1 cm × 2 cm × 0.1 cm) (*n* = 3) was measured by a SurPASS Electrokinetic Analyzer (Anton Paar, France). KCl (0.001 M) was used and neutralized to pH 7.4 by 0.1 M HCl or NaOH to simulate the situation in vivo.

### Adsorbed protein removal by different surfactants

AAPT samples were divided into four groups (*n* = 4) and placed in 24-well plates, and 1 ml of fetal bovine serum (FBS, HyClone, Thermo Fisher Scientific, Logan, UT, USA) was added per well. After incubation at 37 °C in a 5% CO_2_ atmosphere for 1 h, samples were gently rinsed three times with 1 ml of PBS to remove residual FBS and loosely bound proteins and then transferred to new 24-well plates. Three hundred microliters of 5% sodium dodecyl sulfate (SDS, Sigma, St. Louis, MO, USA), 2% SDS, 0.2% Triton X-100 (Sigma) or 0.1% Tween 20 (Guangzhou Chemical Reagent, China) was added per group. The samples were incubated in an ultrasonic bath for 30 min to detach any adsorbed proteins. The protein concentration in solution was quantitatively analyzed by BCA measurements (BCA Protein Assay Kit, Pierce, Thermo Fisher Scientific, USA) according to the manufacturer’s instructions. A mixture of 25 μl of protein sample and 200 μl of working reagent was incubated at 37 °C for 30 min. The absorbance of the mixture was measured by a microplate reader (Tecan, Männedorf, Switzerland) at *λ* 562 nm. The amounts of the adsorbed total proteins were calculated according to the calibrated curve with BSA as the standard. The surfactant corresponding to the highest amount of protein was used to elute the adsorbed protein in the following experiments.

### Serum protein adsorption kinetics

Each of the three groups, i.e., AAPT, NTPT and NTDT, contained six subgroups. Samples (*n* = 4 of each subgroup) were placed in wells with 1 ml of FBS and incubated at 37 °C in a 5% CO_2_ atmosphere for 5, 15, 30, 60, 90 and 120 min. After each incubation period, the FBS was removed, and the samples were gently rinsed three times with PBS and then transferred to new 24-well plates. The samples were immersed in 300 μl of selected surfactant and incubated in an ultrasonic bath for 30 min. The protein concentration was also quantitatively analyzed by the BCA method.

### Thickness of the protein layer

AAPT, NTPT and NTDT samples (*n* = 4) placed in 24-well plates with 1 ml of FBS were incubated at 37 °C in a 5% CO_2_ atmosphere for 1 h. Then, the samples were gently rinsed with PBS three times, followed by evaporation at room temperature. The thickness of the protein layer on each sample was measured by ellipsometry as a function of protein adsorption (Horiba, Montpellier, France), with monitoring of the variations in the ellipsometric angles ∆ and ψ. The ellipsometric measurements were performed at 45° and *λ* 450 nm with a 650 eV xenon light source.

### Components of adsorbed proteins

AAPT, NTPT and NTDT samples (*n* = 4) were placed in 24-well plates with 1 ml of FBS and incubated at 37 °C in a 5% CO_2_ atmosphere for 1 h. Then, the samples were gently rinsed with PBS three times. Protein was collected ultrasonically from sample surfaces. Then, the protein solution was mixed with 5× loading buffer and degraded at 99 °C for 5 min. An equal volume of protein mixture was separated by SDS-polyacrylamide gel electrophoresis (Beyotime, Haimen, China) through a 5% stacking gel and a 10% separating gel in a mini-electrophoresis system (Bio–Rad, Hercules, CA, USA). The gel was run at 80 V until the front line crossed the stacking gel zone. The gel was gently rinsed three times with 50 ml of ddH_2_O for 5 min while shaking. After the gel was washed, 20 ml of Coomassie brilliant blue (Beyotime) was added, and the gel was incubated at 23 °C for 1 h while shaking and then destained in ddH_2_O overnight at 4 °C. Finally, the gel was visualized by a gel imaging system. The intensity of the protein bands was calculated with ImageJ software.

### Bacterial suspensions

The abundant oral colonizer *S. mutans* was chosen in the present study. *S. mutans* (UA159) was grown at 37 °C on a brain heart infusion (BHI) (Huankai, Guangdong, China) agar plate. A single colony was inoculated into 5 ml of BHI broth at 37 °C and cultured overnight. The bacteria were collected by centrifugation at 5000 rpm for 5 min and washed twice with fresh BHI broth. The bacterial pellets were resuspended in BHI broth with 1% sucrose to 1 × 10^6^ CFU/ml.

### Serum precoating

AAPT, NTPT and NTDT samples (*n* = 16) were placed in 24-well plates, after which 1 ml of FBS per well was added for half of the samples. Samples were incubated at 37 °C for 1 h. Then, the FBS was removed, and the samples were gently rinsed with ddH_2_O three times. The nonprecoated samples (AAPT, NTPT and NTDT) and those precoated with FBS (AAPT-P, NTPT-P, NTDT-P) were used in the following experiments.

### SEM

AAPT, NTPT, NTDT and AAPT-P, NTPT-P and NTDT-P samples (*n* = 4) were incubated in 1 ml of an *S. mutans* cell suspension at 37 °C for 1 and 24 h. All samples were rinsed with ddH_2_O three times and fixed with 2.5% glutaraldehyde at 4 °C overnight. The samples were dehydrated through a series of ethanol (25, 50, 75, 95 and 100 v/v%) washes, desiccated by critical-point drying (HCP-2, Hitachi, Tokyo, Japan) and sputter-coated with platinum (E102, Hitachi) for 120 s.

### Confocal laser scanning microscopy (CLSM)

AAPT, NTPT, NTDT, AAPT-P, NTPT-P and NTDT-P samples (*n* = 4) were incubated in 1 ml of an *S. mutans* cell suspension at 37 °C for 1 and 24 h. All samples were rinsed with ddH_2_O three times, stained with the BacLight^TM^ Live/Dead Bacterial Viability Kit for 15 min and analyzed by confocal laser scanning microscopy. 3D image reconstruction was carried out from 30 Z stacks.

### Quantification of bacterial adhesion

AAPT, NTPT, NTDT, AAPT-P, NTPT-P and NTDT-P samples (*n* = 4) were incubated in 1 ml of an *S. mutans* cell suspension at 37 °C for 1 and 24 h. All samples were rinsed with ddH_2_O three times, stained with 0.1% crystal violet solution for 15 min after fixation, washed with ddH_2_O and dried. The samples were rinsed until the excess crystal violet was removed. All samples were extracted in 1 ml of 95% ethanol and quantified by measuring the absorbance at 595 nm (*A*_595_).

### Statistical analysis

Differences in water contact angle, SSA, zeta potential, protein concentration, intensity of protein bands and *A*_595_ were analyzed with one-way ANOVA followed by a least significant difference test. Statistical analyses were performed with SPSS 23. Differences and parameters were regarded as statistically significant at the relevant level of 0.05.

## Results

### Surface characteristics of Ti with or without surface treatments

**Fig. 1:**
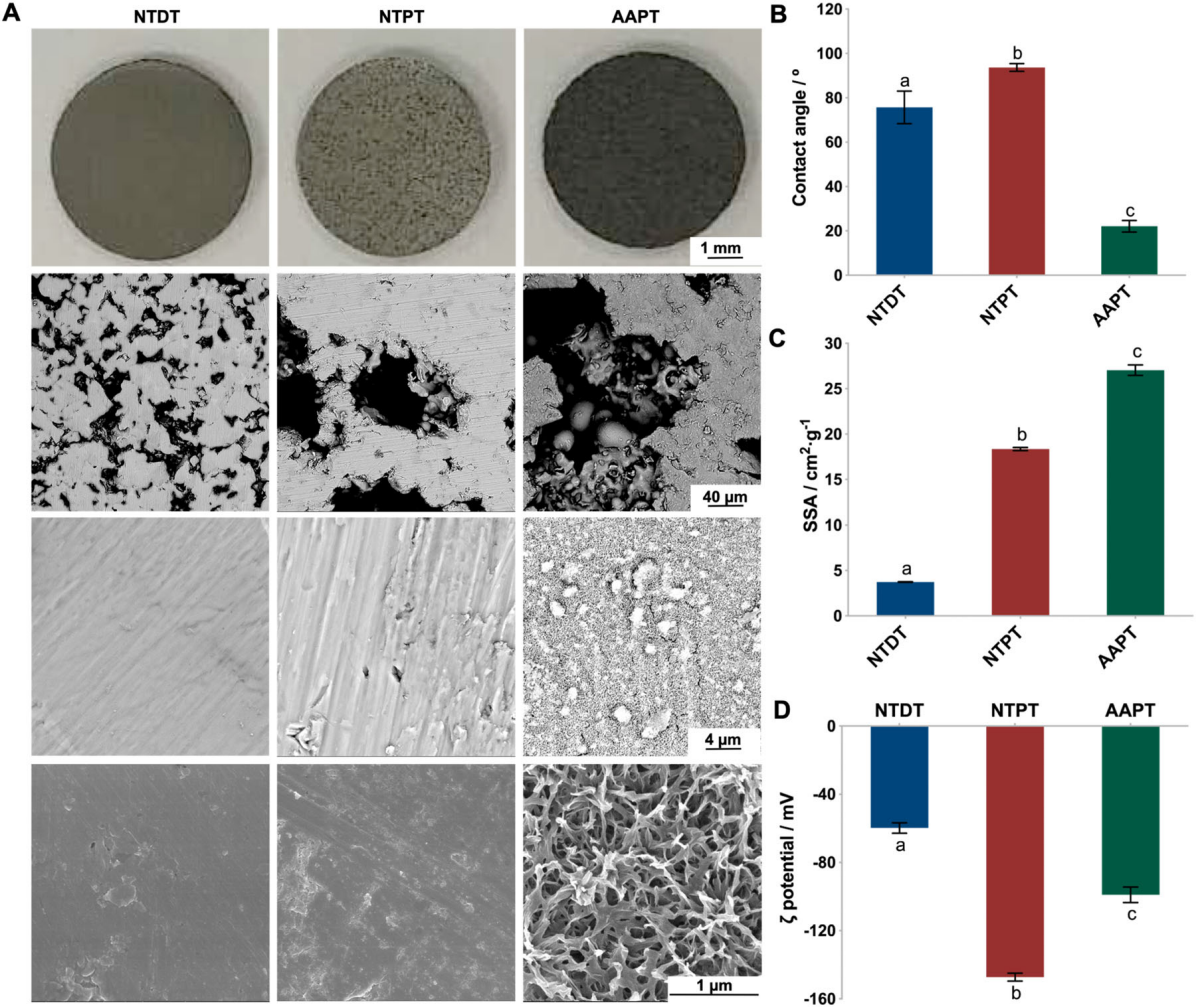
Surface characteristics of Ti with or without surface treatment. **A** The surface topography of NTDT, NTPT and AAPT was observed by SEM, with AAPT showing needle-like submicropores and nanopores (*n* = 3). **B** Contact angles were measured by the sessile-drop test (*n* = 6). **C** SSAs of samples were determined by the BET method (*n* = 3). The results showed that AAPT adsorbed more nitrogen than NTPT and NTDT after acid-alkali treatment. **D** Zeta potentials were measured at pH 7.4 to simulate the situation in vivo (*n* = 3). Different letters denote significant differences between/among groups (same as below)

Generally, the NTDT surface appeared smooth, since parallel scratches were clearly outlined, and some sporadic micropores could be observed. In comparison, the rougher surface of AAPT with homogeneous and regular micropores was apparent, showing needle-like submicropores and nanopores at higher magnification (Fig. 1A). After acid-alkali treatment, the contact angle of AAPT was 22.1° ± 2.6, much smaller than 93.7° ± 1.7 for NTPT and 75.7° ± 1.7 for NTDT (*p* < 0.001) (Fig. 1B). Moreover, AAPT adsorbed more nitrogen than NTPT and NTDT (*p* < 0.001). In the nontreated sample, porous Ti showed more nitrogen adsorption than NTDT (*p* < 0.001) (Fig. 1C). After acid-alkali treatment of porous samples, the zeta potential shifted gradually from −147.26 mV (NTPT) to −98.98 mV (AAPT) (*p* < 0.001). The dense sample showed the lowest zeta potential of −59.83 mV ± 3.05 (*p* < 0.001) (Fig. 1D).

### Serum protein adsorption of Ti with or without surface treatment

**Fig. 2:**
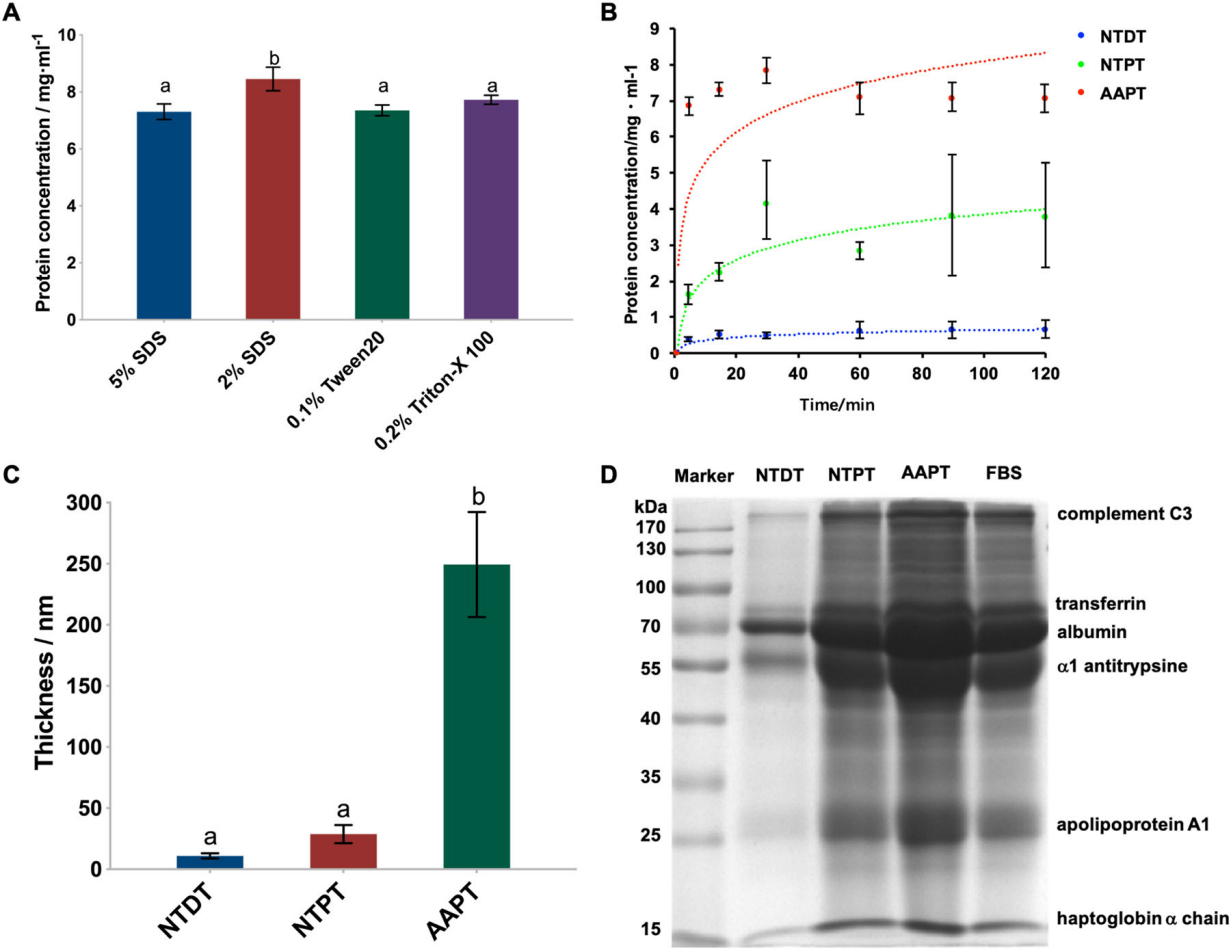
Serum protein adsorption of Ti with or without surface treatment. **A** Detached protein concentration obtained with different surfactants. The results showed that 2% SDS was most efficient among the applied solvents and was used to elute the adsorbed proteins in the following experiments (*n* = 4). **B** The adsorbed protein amount in each group at each time point was quantitatively analyzed by the BCA method (*n* = 4). The results showed that AAPT adsorbed more serum protein than NTPT and NTDT (*p* < 0.001). **C** Thickness of the adsorbed protein layer on each group measured as a function of protein adsorption, showing that the protein layer on the AAPT sample was thicker than that on the other two samples (*n* = 4). **D** SDS–PAGE patterns of the adsorbed proteins were visualized by a gel imaging system (*n* = 4). The results showed that more protein bands were detected on NTPT and AAPT than on NTDT

The protein removal effectiveness assay showed that 2% SDS was most efficient among the applied solvents, with the highest detached protein concentration obtained from AAPT samples (*p* < 0.001), and this concentration was used to elute the adsorbed proteins in the following experiments (Fig. 2A). In general, the adsorption kinetics of serum proteins on porous Ti samples largely increased and fit an approximately logarithmic relationship. At each time point in the incubation period, AAPT adsorbed more serum protein than NTPT and NTDT (*p* < 0.001). Protein adsorption reached equilibrium at 90 min. The dense samples did not appear to be able to adsorb serum protein. The cumulative protein amount on these samples was much less than that on porous Ti, increasing transiently and then plateauing after 30 min. Changes in adsorbed proteins on dense Ti over time did not correlate with incubation (Fig. 2B). The protein layer on the AAPT sample was thicker than that on the other two samples (*p* < 0.001) and was related to the surface structure. The thicknesses of the protein layers on nontreated dense and porous samples did not differ from each other (*p* = 0.08) (Fig. 2C). The main proteins adsorbed on all samples were albumin, α1 antitrypsin, transferrin, apolipoprotein A1, complement C3 and haptoglobin α1 chain. More protein bands were detected on NTPT and AAPT than on NTDT (Fig. 2D). Compared with the profiles of FBS and markers, the main protein components in FBS were adsorbed on NTPT and AAPT, while only some adhered to NTDT. The intensity of the bands indicated greater protein adsorption on precoated samples than on nonprecoated samples (Table 1).

**Table 1:** Intensities of protein bands of the adsorbed proteins on NTDT, NTPT and AAPT

Proteins	MW (kDa)	NTDT (NTDT/FBS)	NTPT (NTPT/FBS)	AAPT (AAPT/FBS)
Albumin	66	192.30 ± 1.00 (0.77)	239.09 ± 0.86 (0.95)	303.33 ± 1.59 (1.21)
α1 antitrypsine	54	130.30 ± 0.19 (0.55)	238.53 ± 0.36 (1.01)	330.94 ± 0.29 (1.41)
Transferrin	77	64.32 ± 0.12 (0.55)	116.77 ± 0.20 (0.95)	181.55 ± 0.17 (1.48)
Complement C3	195	21.98 ± 0.02 (0.22)	86.53 ± 0.11 (0.87)	144.07 ± 0.21 (1.45)
Apolipoprotein A1	28	41.86 ± 0.03 (0.16)	259.89 ± 0.09 (1.10)	396.78 ± 0.11 (1.68)
Haptoglobin α chain	15	17.77 ± 0.01 (0.21)	111.69 ± 0.05 (1.32)	139.83 ± 0.10 (1.65)

### Bacterial adhesion after 1 and 24 h of incubation on Ti with or without serum precoating

**Fig. 3:**
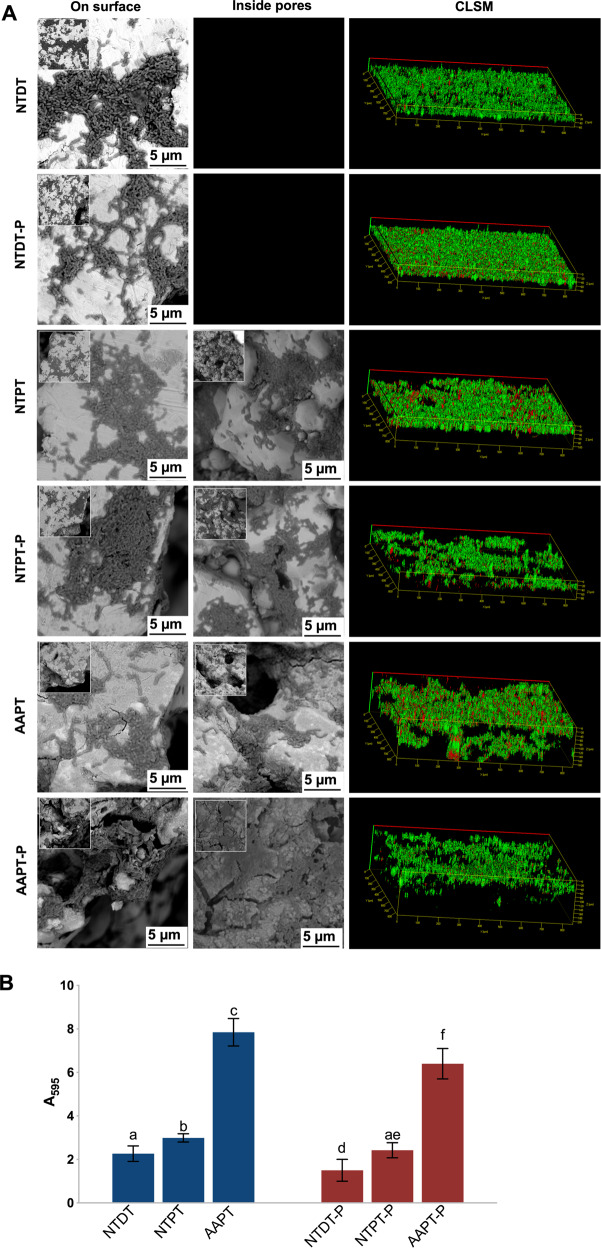
*S. mutans* adhesion shortly after the start of the 1 h incubation period on Ti with or without serum precoating. **A** SEM and CLSM images showed the morphology of bacterial cells adhered to NTDT, NTDT-P, NTPT, NTPT-P, AAPT, and AAPT-P. *S. mutans* showed a normal shape and structure (long chain). Some scattered *S. mutans* and small colonies were observed after 1 h of incubation. Bacterial aggregation on AAPT and AAPT-P seemed to be more evident than on NTPT and NTDT as well as on NTPT-P and NTDT-P. **B** Quantification of bacterial adhesion in each group was performed by measuring the absorbance at 595 nm (*n* = 4). The results showed that bacteria adhered more to porous samples than to dense samples (*p* < 0.05) and adhered more to nonprecoated samples than to protein-precoated samples (*p* < 0.05)

**Fig. 4:**
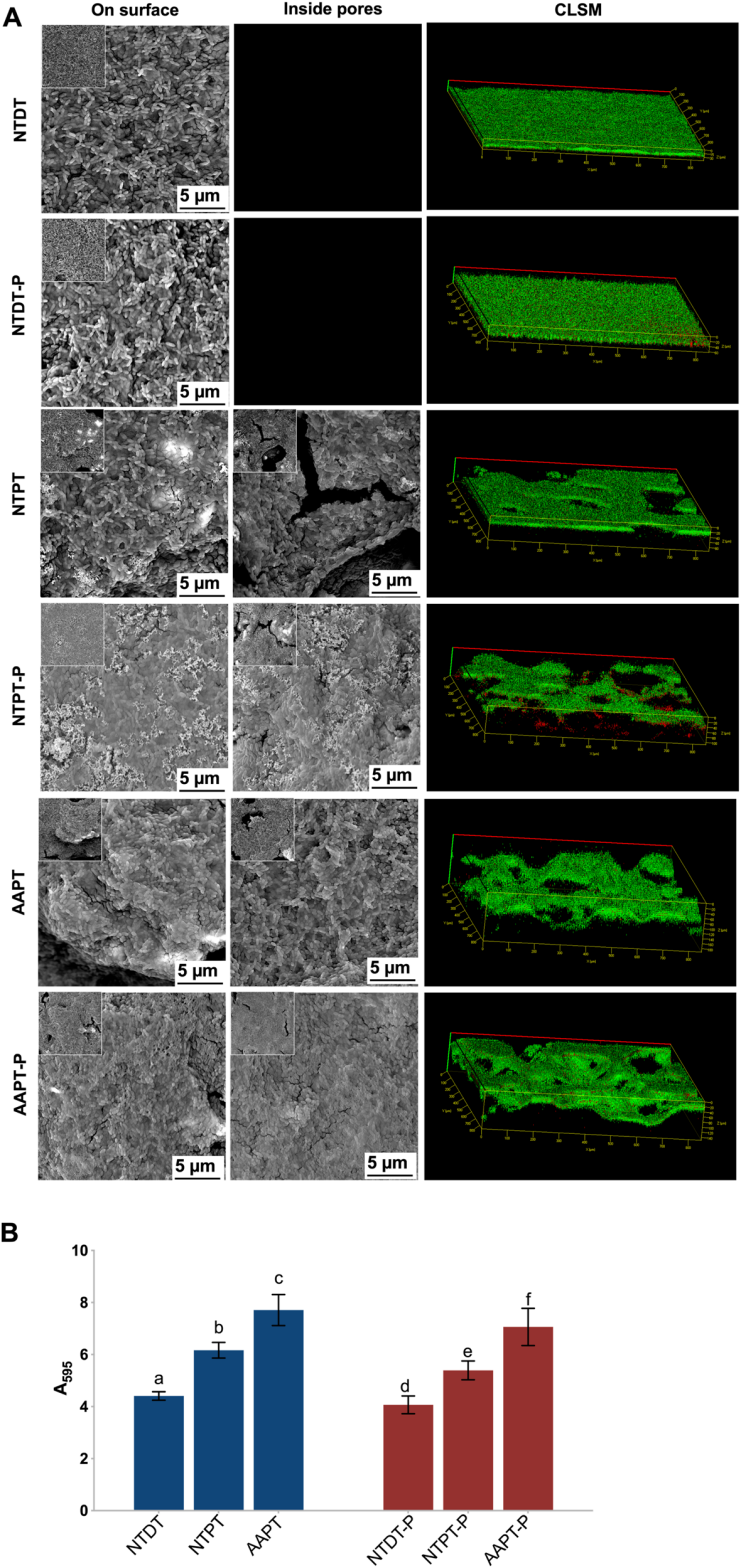
*S. mutans* adhesion was finally observed at the endpoint (24 h) of incubation on Ti with or without serum precoating. **A** SEM and CLSM images showed the morphology of bacterial adhesion on NTDT, NTDT-P, NTPT, NTPT-P, AAPT, and AAPT-P. The results showed that more *S. mutans* cells clustered to form biofilms coating all sample surfaces. Bacterial aggregation on AAPT and AAPT-P seemed to be more evident than on NTPT and NTDT as well as on NTPT-P and NTDT-P. **B** Quantification of bacterial adhesion in each group was performed by measuring the absorbance at 595 nm (*n* = 4). The results showed that bacteria adhered more to porous samples than to dense samples (*p* < 0.05). It was also evident that protein coating tended to prevent the bacteria from adhering to material surfaces

*S. mutans* showed a normal shape and structure (long chain). Some scattered *S. mutans* cells and small colonies were observed after 1 h of incubation. Since the incubation lasted for 24 h, more *S. mutans* clustered to form biofilms coating all sample surfaces. Bacterial aggregation on AAPT and AAPT-P seemed to be more evident than on NTPT and NTDT as well as on NTPT-P and NTDT-P (Figs. 3A and 4A). Shortly after the start (1 h) and finally at the endpoint (24 h) of incubation, bacteria, in general, adhered more to porous samples than to dense samples (*p* < 0.05). It was also evident that protein coating tended to prevent the bacteria from adhering to material surfaces, i.e., more *S. mutans* adhered to nonprecoated samples than to protein-precoated samples (Figs. 3B and 4B).

## Discussion

Surface treatment of biomaterials is the most common means of affecting protein adsorption and cellular behavior in situ. The combination of acid-alkali treatment facilitates the formation of nanosized pores on the basis of the microsized pores of porous Ti [23]. Protein adsorption onto porous materials is based on the micropores of porous scaffolds [24]. The introduction of nanopores increases surface roughness and favors protein adsorption [25]. In the present results, acid-alkali-treated porous samples showed the highest adsorption of serum protein, consistent with results from a previous related study that found that acid-alkali treatment rendered Ti hydrophilic [26]. It has been suggested that surface treatment removes contaminants from the material surface and gives rise to the hydrophilicity of Ti [27, 28].

Ti is chemically very active and has a strong tendency to passivate. Any commercial Ti implant exposed to air in the clinic is inevitably covered by a thin and dense layer of TiO_2_. The in vivo layer attracts H^+^, leaving OH^−^ and generating a negatively charged surface that discourages protein adsorption [29]. Removal of the layer adjusts the surface potential and is deemed to be effective for protein adsorption. Following acid-alkali treatment, sodium titanate on the superficial layer, via reaction with H_2_O, has been suggested to form Ti–OH, ultimately resulting in a negative zeta potential. Since the zeta potential depends on the chemical composition of the biomaterial surface, the decrease in potential after treatment in the present results should be attributable to the formation of –OH groups on the surface [30].

A feasible method has been suggested whereby surfactants could be used to remove surface-bound protein [31]. Consistent with results from related studies, our results showed that SDS was the most efficient among the applied solvents [32, 33]. With the assistance of ultrasonic treatment [34], 2% SDS was chosen for the following experiments.

It is well known that the pores in porous materials provide more binding sites for proteins and facilitate protein adsorption, since proteins can be trapped inside pores [35]. When proteins inside pores and on the pore walls occupy any available space [3], the amount of protein in the superficial layer of the porous structure does not increase. Our results clearly showed the saturation of protein adsorption on the porous Ti samples. Acid-alkali treatment of Ti pores enhanced the effect, and as expected, the samples adsorbed much more protein. In comparison, proteins hardly seemed to have been adsorbed on the dense sample surfaces without micro- and nanopores. The adsorbed protein amount was consistent with the thickness of the protein layer, and AAPT had a thicker protein layer.

Protein adsorption onto the biomaterial surface is also related to surface polarity. Whether a hydrophobic or a hydrophilic surface favors protein, however, remains controversial [36, 37]. In our results, the treated porous Ti showed clear hydrophilicity and appeared to be most efficient in protein adsorption, consistent with the results of a previous study suggesting that a hydrophilic surface with a nanostructure facilitates protein adsorption [38].

Protein adsorption onto the material surface is also affected by electrostatic interactions. It is well known that most serum proteins are negatively charged under physiological conditions. In the present results, all the samples, whether dense or porous, precoated or nonprecoated, showed negative potentials, yet the proteins were adsorbed onto the surface. It seemed that electrostatic interactions dominated protein-surface interactions only when the surface and the protein were oppositely charged [39]. In our results, the potential of porous samples was much less than that of dense samples, and the porous samples still adsorbed more protein. Apparently, the porous structure on the surface provided a powerful ability to trap and bind proteins, which could compensate for and resist the repulsive electrostatic force. In this sense, protein adsorption on NTPT must overcome more electrostatic resistance than that on AAPT, resulting in less protein on NTPT than on AAPT.

Protein adsorption onto acid-alkali-treated Ti showed no selectivity. The kinds of proteins adsorbed onto NTPT and AAPT did not appear to differ from those of FBS, except for slight differences in relative abundance, which was consistent with the results of related studies [40, 41].

In the present study, AAPT adsorbed much more albumin than NTDT and NTPT, almost 60% and 30%, respectively. The biocompatibility of a material is related to the amount of albumin on its surface [42]. The nanostructure of a material allows an increase in protein unfolding [43, 44], exposing hidden cell-binding sites [45] that can be used as reservoirs for growth factors. It has been suggested that the nanostructure of Ti following acid-alkali treatment might facilitate material biocompatibility with respect to its adsorbed protein layer.

In recent decades, rougher surfaces have been suggested to favor bacterial adhesion [46, 47]. However, although it facilitates the formation of a protein film layer, surface modification to form micro- and nanostructures on the biomaterial raises concerns regarding the increase in bacterial adhesion. In our results, the porous structure of all the samples was covered by a protein layer. Furthermore, the precoated protein samples prevented the bacteria from adhering, regardless of whether the samples were dense or porous. Albumin seemed to inhibit bacterial adhesion [48]. The effects of bacterial adhesion and colonization should be compensated for by other factors. When the surface roughness (Ra) was less than 0.2 µm, it had no effect on bacterial adhesion. [49] Surface polarity also affects bacterial affinity, and a hydrophobic surface allows higher bacterial colonization [50]. In this study, all the porous samples became much more hydrophilic after acid-alkali treatment. The electrostatic interaction in the study also strengthened the protein-precoated porous samples to resist bacterial adhesion, since serum proteins are negatively charged and bacteria in aqueous suspension are almost always negatively charged [51].

## Conclusion

Acid-alkali treatment facilitated protein adsorption onto porous Ti, and the protein coating tended to prevent bacteria from adhering. Lower-MW proteins showed higher affinity to porous Ti. The modified surface became much more hydrophilic and exhibited a lower zeta potential.
